# Measurements of the flow of a liquid–solid mixture/suspension through a segmented orifice

**DOI:** 10.1038/s41598-023-50737-6

**Published:** 2024-01-02

**Authors:** Marcin Heronimczak, Andrzej Mrowiec, Mariusz Rząsa, Krzysztof Koszela

**Affiliations:** 1 Calisia University, Polytechnic Faculty, 62-800 Kalisz, Poland; 2 Opole University of Technology, Faculty of Electrical Engineering, Automatic Control and Informatics, 45-758 Opole, Poland; 3 Department of Biosystems Engineering, Poznan University of Life Sciences, 60-637 Poznan, Poland

**Keywords:** Mechanical engineering, Fluid dynamics

## Abstract

The paper attempts to solve the metrological problem that occurs when measuring the intensity of a flowing fluid with suspended solids with densities greater and less than the density of the fluid. The issue of the possibility of self-cleaning of a prototype variant of a segmented orifice from floating solid particles forming mixture/suspensions is discussed. For spherical particles of solids calculations have been made to allow for determining a borderline between their floating and entrainment by the flow, based on dimensionless numbers: Archimedes number and Reynolds number. Experimental tests and CFD simulations were conducted with a variable flow determined by Reynolds number for comparable segmental orifices with orifice module m = 0.102. Flow characteristics were plotted. Based on the results obtained from numerical simulations, positive influence of the inclination of skew segmental orifice downflow plane was presented. The results obtained from the study are a guideline for planning further studies to expand the knowledge of segmented orifices with inclined inflow plane.

## Introduction

The flow of fluid–fluid or fluid–solid mixtures often occurs in industrial processes. Therefore, knowledge of continuous phase of fluid (gas or fluid) in a mixture is desired when designing, manufacturing and using machines or equipment in the such processes. Flow of mixtures that occurs in technological processes can be categorized into: sedimentation, fluidization, pneumatic transport, and hydraulic transport. Source literature presents fluid mixtures in the form of water–oil or oil–water for different ratios in the studied mixture^1^.

However, considering only the fluid–solid mixture itself, depending on particle density of fluid and solids, we are dealing with unbounded movement of settling or entrained particles, including their forced movement on curvilinear paths. It is problematic to describe such complex problems with mathematical equations easily^2^. The occurring unbounded velocity of falling or entrainment depends on many factors. However, source literature mainly presents the problem of solids settling in a fluid. With contemporary state of knowledge and measurement instruments used, there is no problem in determining the velocity and trajectory of movement of individual particles. Yet, theoretical bases existing at this point are necessary for describing movement in the case of grouping of solids^3^. Such approach justifies further search for research methods and theoretical solutions in the case of movement of fluid–solid mixtures in horizontal pipes. If we limit the problem to hydraulic transport only, we will deal with fluid–solid type mixtures. This means of transport mainly occurs in construction and extractive industry^4^. It is used for transporting loose materials that do not dissolve in water, such as coal, ore or sand. In terms of transporting materials in horizontal pipes, literature presents a simplified model of behavior of particles in a solid–fluid mixture in transitory and turbulent flow (equation of motion, lift and drag coefficients)^5^.

Flow structure in a mixture depends on the ratio of fluid density to solid phase density, including its concentration, but also on the velocity of the flowing fluid. When solid particles are of similar density to the fluid, they move along with it with similar velocity to the flow, creating a uniform structure in its entire volume. Such flow type occurs for very small particles, usually smaller than 0.15 mm, and collisions among them are very rare. When particles of the solid phase are larger or their density is higher than the density of the fluid, the flow becomes asymmetric relative to the axis of the horizontal flow channel, the particles move in its bottom part^6^. When the velocity of the flowing fluid is reduced, the asymmetry increases, causing a flow of mixture with a moving bottom deposit. What happens is that solid particles roll on the bottom of the horizontal pipeline with much lower velocity than the flowing fluid^7,8^.

In industrial technological processes, where constant measurement of the volumetric flow rate is required, such mixtures create metrological problems even at low concentration of solid particles in the fluid (e.g. contamination of furnace oil with sand). For this reason, mechanical flow meters are useless for measuring, since rotating measurement elements become damaged due to concentration of solid particles in the metering mechanism. Among non-invasive flow meters, for measuring the volumetric flow rate of a fluid–solid suspension, we use ultrasonic and electromagnetic flow meters^9,10^. However, the most popularized, cheap and reliable instruments for measuring flow are constriction flow meters. They utilize sudden constriction of the pipeline that the flowing fluid encounters. In the constriction, rapid increase in flow velocity occurs, resulting in creating a difference in static pressure before and after the constriction. The following venturi are used as reducers for measuring the flow: venturi tubes, nozzles and orifices^11,12^. In spite of their flaws, among which are: constant pressure loss with fluid flow and low rangeability (4:1), orifices are popular in measuring flow, due to their simple structure and reliability in use. Among standardized, known structures of measuring orifices, the standard (centrical) orifice has been adopted as basic. It has a typical accuracy of 1.5–2%^13^. Conducted experimental tests has revealed that during the flow of contaminated fluids such as fluid–solid suspension, the geometry of the pipeline and inlet edge of the orifice changes through deposition of solids, causing a change in flow kinematics^14^. The example proves that when flow requirements for fluids, specified in the ISO 5167-1 standard^15^ cannot be met, there is a possibility to use non-standard orifices. They include such orifices, as: quadrant orifice (used particularly for flows with low Reynolds numbers), eccentric orifice, or segmental orifice. We use the latter two types of orifices mainly for the flow of liquids contaminated with solids in which the density of the contaminant particle is greater or less than the density of the fluid. In this case, we adjust the orifice opening so that the impurities can flow freely through the measuring orifice^16–18^. Particles of inclusions with density ${\uprho }_{{\text{cz}}}>{\uprho }$ (particle heavier than the fluid) are transported in the lower part of the pipeline, so the through hole of an eccentric or segmental orifice is placed so that it is tangent to the lower part of the pipeline. The inclusion particles with a density lower than that of the fluid ${\uprho }_{{\text{cz}}}<{\uprho }$, according to the buoyancy force, move in the upper part of the pipeline along with the fluid. In this situation, the through hole of the measuring orifice is placed in the upper part of the pipeline, which ensures the free flow of impurities in the measured fluid. This avoids the formation of deposits that restrict the flow in the pipeline in front of the venturi. Placing on the opposite side from the through hole of the orifice, impulse holes for taking pressure, eliminates the risk of plugging (clogging) of the holes, which can cause erroneous measurement of the difference of accumulated pressure^14,15^.

Source literature contains few articles on flow through segmental orifices. One of them presents simulations of large vortexes (the SBES model) during flow through segmental orifices with different constriction. The obtained results of flow were verified by measurement using the ultrasonic flow meter and the Laser Doppler Anemometric flow meter (LDA)^19^. The influence of distortion caused by sudden change in flow direction due to inserting a 90° elbow in the flow system before the segmental orifice was presented in the form of experimental tests. The influence of distance between the elbow and the segmental orifice on the accuracy of flow rate measurement^20^. Whereas, article^21^ presents flow study of mixtures with various concentration, in the form of emulsion that combines water and special oil dissolved in. Study was conducted for two constriction coefficients $\beta$ equal to 0.3 and 0.5. It was found that the value for flow coefficient decreases whenever the constriction coefficient increases. In another article the authors presented experimental and numerical tests for a prototype flow meter with an segmental orifice inclined by an angle of 60° against the axis of the pipeline for constriction $\beta =0.5$. The authors assessed the value of flow coefficient in a developing turbulent flow^22^.

Metrological problems are encountered when measuring the stream of the liquid phase contaminated with a mixture of solid particles of different densities. Particles of solids with a higher density (${\uprho }_{{\text{cz}}})$ than the density of the liquid ($\uprho$) flow freely through the orifice constriction set at the bottom of the pipeline. On the other hand, particles with a density lower than that of the liquid (${\uprho }_{{\text{cz}}}>{\uprho }$) accumulate in the upper part of the pipeline in front of the orifice disrupting the flow and the measurement of the differential pressure value being an indirect measurement in determining the flux of the flowing liquid–solid mixture. By inclining the inflow plane of the segmental orifice by angle $\alpha^\circ$ it was assumed that the new velocity distribution will allow the flowing fluid to entrain more solid particles with density of ${\rho }_{cz}<{\rho }$ from area located before the measurement orifice. The presented assumption was defined as ‘self-purging process’ of the measurement orifice from deposits that settle before it.

In order to solve the problem in the top part of the pipeline, the article proposes a flow meter based on an area reducer in the form of a skew segmental orifice that enables self-purging. For such a prototype of a measurement orifice, numerical study of ‘self-purging’ have been conducted for a flowing mixture of fluid and solids with lower density than the fluid.

## Materials and methods

**Figure 1:**
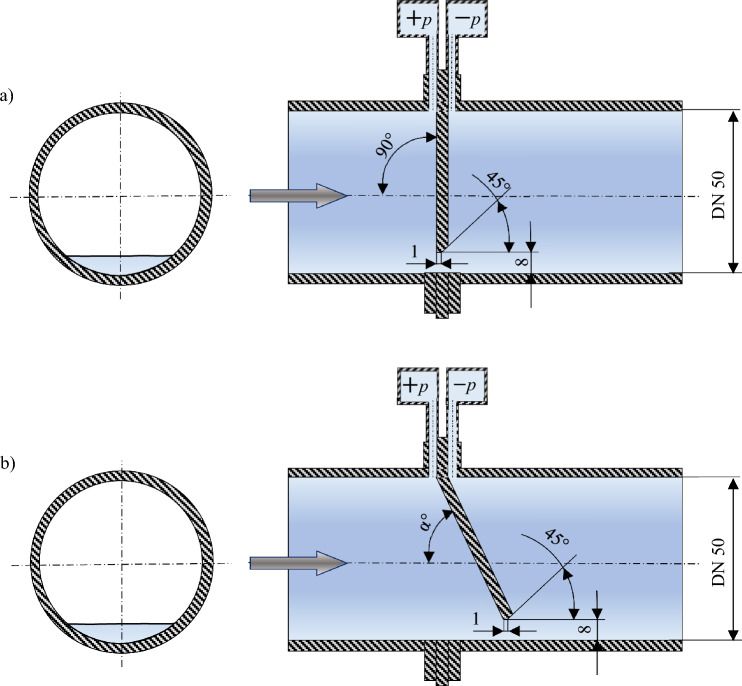
The studied object: (**a**) segmental orifice (m = 0.102); (**b**) segmental orifice (m = 0.102) with inclined plane [own work].

Experimental tests and CFD numerical simulations have been conducted for a segmental orifice with module $m=0.102$ made according to standard^17^ and on a prototype segmental orifice wich inflow plane inclined by angle α in line with the flow direction (Fig. 1).

Results of conducted experimental measurements have produced flow characteristics through a segmental orifice and segmental orifices with inflow plane inclined by angle $\alpha =70^\circ$. They have been presented as mass flow of the flowing fluid in a function of build-up value of static pressure on a measurement orifice during flow.

To theoretically determine the flux of a flowing incompressible, non-viscous fluid, we use the stream continuity equation in conjunction with Bernulli's equation. The stream continuity equation for a flowing incompressible fluid is given by the relation:

1 $${q}_{v}={{F}_{1}\cdot \vartheta }_{1}={{F}_{2}\cdot \vartheta }_{2}$$

The point of constriction of the orifice $({F}_{1}>{F}_{2})$, there is an increase in the velocity of the fluid (${\vartheta }_{2}>{\vartheta }_{1}$). According to the principle of conservation of energy (Bernoulli's law), when there is an increase in kinetic energy (increase in velocity), there is a decrease in potential energy (decrease in pressure), so:

2 $$\frac{\rho {\vartheta }_{1}^{2}}{2}+{p}_{1}=\frac{\rho {\vartheta }_{2}^{2}}{2}+{p}_{2}=const$$

After the simplifications adopted and the transformations carried out, the expression for the theoretical volume flux ${q}_{V}$ was obtained:

3 $${q}_{V}=\frac{{F}_{2}}{\sqrt{1-{m}^{2}}}\cdot \sqrt{\frac{2({p}_{1}-{p}_{2})}{\rho }}$$

However, in practice, the value of the flux of the flowing fluid determined from the above relationship differs from the actual flux. This is a consequence of the simplifications that were adopted in the mathematical attempt to describe the physical phenomena occurring during the flow of fluid through the measuring venturi. For this reason, the flow coefficient C (proportionality factor) was introduced into the equation determining the actual value of the flowing fluid stream, the equation takes the form:

4 $${q}_{v}=\frac{C\cdot {F}_{2}}{\sqrt{1-{m}^{2}}}\cdot \sqrt{\frac{2\cdot \Delta p}{\rho }}$$

Results obtained from experimental tests have been subjected to further calculative analysis to determine type B uncertainty of the conducted measurements of flow and pressure build-up on the orifice. The relative standard uncertainty of the measurement of the pressure pile-up at the measuring orifice is equal to:

5 $$\frac{{u(\Delta p)}}{{\Delta p}} = \frac{{\sqrt {S_{{\Delta p}}^{{2}} + \left( {\frac{{\Delta g_{{(\Delta p)}} }}{{\sqrt 3 }}} \right)^{2} } }}{{\Delta p}} \cdot 100\%$$

From relation (5), the relative expanded uncertainty of the measurement of the pressure buildup $\Delta p$ at the measuring orifice was determined as $U(\Delta p)/\Delta p$ (with the assumed confidence level $p=\mathrm{0,95}$ and the expansion factor ${k}_{p}=2$), which can be written in the form of the relation:

6 $$\frac{U(\Delta p)}{\Delta p}={k}_{p}\cdot \frac{u(\Delta p)}{\Delta p}$$

We determine the relative standard uncertainty of the measurement of the mass flux flowing through the measuring orifice from Eq. (7):

7 $$\frac{u({q}_{m})}{{q}_{m}}=\sqrt{{\left(\frac{u({q}_{v})}{{q}_{v}}\right)}^{2}+{\left(\frac{u\left(\rho \right)}{\rho }\right)}^{2}}$$

where:

- the relative standard uncertainty of the flux measurement is:
  8 $$\frac{{u(q_{v} )}}{{q_{v} }} = \frac{{\sqrt {S_{{q_{v} }}^{{2}} + \left( {\frac{{\Delta g_{{(q_{v} )}} }}{{\sqrt 3 }}} \right)^{2} } }}{{q_{V} }} \cdot 100\%$$
- the relative standard uncertainty of the determination of water density, it was determined using the table of water density as a function of temperature from the relationship:
  9 $$\frac{u({\rho })}{\rho }=\frac{\frac{{\Delta }_{\rho }}{\sqrt{3}}}{\rho }\cdot 100\%$$

In the measurement series carried out, the temperature of the flowing water changed in the range up 2 °C, which corresponds to the relative standard uncertainty of its determination

$$\frac{u({\rho })}{\rho }=0.05\%.$$

From relations (7–9), the assumed relative expanded uncertainty of the measurement of the mass flux ${q}_{m}$ flowing through the measuring orifice was determined as $U({q}_{m})/{q}_{m}$ (with the assumed confidence level and expansion factor ${k}_{p}=2$), which can be written in the form of the relation:

10 $$\frac{U({q}_{m})}{{q}_{m}}={k}_{p}\cdot \frac{u({q}_{m})}{{q}_{m}}$$

The final stage will use graphic velocity distribution, from numerical simulations, before the measurement orifice, for different angles of inclination of the downflow plane, to analyze the velocity of entrainment of a single particle. Based on the analysis, a reduction coefficient for adverse, blind spot in the area of the pulsive opening before the measurement orifice will be determined.

### CFD numerical tests

Numerical tests used in fluid mechanics (CFD) has become a very popular tool used in the development new industrial measurement systems. The CFD numerical simulation has been performed in ANSYS FLUENT 2020R1 software where turbulent models $k-\omega BSL$ (2 equations) and $TransitionSST$ (4 equations) have been subjected to selection analysis. Both presented models are an extension of model *k*–*ω* standard^23,24^.

Model *k*–*ω*BSL has been selected to conduct numerical flow tests in the work^25^ where validation from several turbulent models for similar flow conditions was performed. The adopted model *k*–*ω*BSL, allows for using the benefits of the known calculation model *k*–*ω* in internal area of the boundary layer, whereas in the external part modelling is performed in model *k*–*ε* which, excluding boundary layer, does a much better job. The model has been described with Eqs. (11, 12):

11 $$\frac{\partial }{\partial t}\left(\rho k\right)+\frac{\partial }{\partial {x}_{i}}\left(\rho k{u}_{i}\right)=\frac{\partial }{\partial {x}_{j}}\left({\Gamma }_{k}\frac{\partial k}{\partial {x}_{j}}\right)+{G}_{k}-{Y}_{k}+{S}_{k}+{G}_{b}$$

12 $$\frac{\partial }{\partial t}\left(\rho \omega \right)+\frac{\partial }{\partial {x}_{i}}\left(\rho \omega {u}_{i}\right)=\frac{\partial }{\partial {x}_{j}}\left({\Gamma }_{\omega }\frac{\partial \omega }{\partial {x}_{j}}\right)+{G}_{\omega }-{Y}_{\omega }+{D}_{\omega }+{S}_{\omega }+{G}_{\omega b}$$

Equation (11) is identical to that in model *k*–*ω* standard, while in the energy transport equation $\omega$ (12) the term ${D}_{\omega }$ has been added, which is a component of cross diffusion that allows to connect model $k-\omega$ with $k-\varepsilon$.

Source literature reveals that the model $TransitionSST$ is the most accurate and reliable in calculating flow, compared to known models from $k-\omega$ group. It allows for a smooth transition from the turbulent model $k-\omega$ into the turbulent model $k-\varepsilon$ which is predisposed for numerical calculations in the main flow. To determine the course of kinematic energy transport $\omega$ in the model $TransitionSST$, an equation from the model $k-\omega standard$ has also been used (13):

13 $$\frac{\partial }{\partial t}\left(\rho \omega \right)+\frac{\partial }{\partial {x}_{i}}\left(\rho \omega {u}_{i}\right)=\frac{\partial }{\partial {x}_{j}}\left({\Gamma }_{\omega }\frac{\partial \omega }{\partial {x}_{j}}\right)+{G}_{\omega }-{Y}_{\omega }+{S}_{\omega }+{G}_{\omega b}$$

The equation for turbulent kinematic energy *k* has been modified to^26,27^ (Eq. 14):

14 $$\frac{\partial }{\partial t}\left(\rho k\right)+\frac{\partial }{\partial {x}_{i}}\left(\rho k{u}_{i}\right)=\frac{\partial }{\partial {x}_{j}}\left({\Gamma }_{k}\frac{\partial k}{\partial {x}_{j}}\right)+{G}_{k}^{*}-{Y}_{k}^{*}+{S}_{k}$$

In this model, Eqs. (13, 14) have been complemented with optional equations for discontinuity transport $\gamma$ (Eq. 15):

15 $$\frac{\partial \left(\rho \gamma \right)}{\partial t}+\frac{\partial \left(\rho {U}_{j}\gamma \right)}{\partial {x}_{j}}={P}_{\gamma 1}-{E}_{\gamma 1}+{P}_{\gamma 2}-{E}_{\gamma 2}+\frac{\partial }{\partial {x}_{j}}\left[\left(\mu +\frac{{\mu }_{t}}{{\sigma }_{\gamma }}\right)\frac{\partial \gamma }{\partial {x}_{j}}\right]$$

It includes transitioning in the boundary layer from laminar flow into turbulent flow, taking account of, apart from the former, transitory momentum thickness transport (Eq. 16):

16 $$\frac{\partial \left(\rho {\widetilde{Re}}_{\theta t}\right)}{\partial t}+\frac{\partial \left(\rho {U}_{j}{\widetilde{Re}}_{\theta t}\right)}{\partial {x}_{j}}={P}_{\theta t}+\frac{\partial }{\partial {x}_{j}}\left[{\sigma }_{\theta t}\left(\mu +{\mu }_{t}\right)\frac{\partial {\widetilde{Re}}_{\theta t}}{\partial {x}_{j}}\right]$$

The above models have a calculative mechanism of transition from model *k*–*ω* in the internal area of the boundary layer to the model *k*–*ε* in its external part. However, they differ in the degree of solving this phenomenon. Detailed information and methods for determining individual parameters in the presented Eqs. (11–16) can be found in the Ansys Fluent 2020R1 software manual^26^.

The necessary computational grid is an important component determining the results and time necessary for conducting numerical simulations^28,29^. Insufficient quality of the computational grid in the conducted numerical simulations will result in erroneous results in the solution of equations during iteration and/or insufficient convergence in *results*. Whereas, a grid built of individual cells which are too small (fine-grained structure) excessively prolongs the time necessary to generate it and the duration of calculations themselves^30–33^

Model domain is being divided into smaller, individual volumes, forming a MOV (finite volume method) type computational grid. To conduct numerical tests, a grid has been developed using the Mosaic Meshing Technology structure^34^. A significant refinement of the grid near the walls has been proposed, allowing for presenting flow phenomena in the boundary layer (transition of the laminar flow into turbulent flow). Vortexes begin to occur in this area, causing energy loss, and areas with accumulation of contaminating particles begin to appear. For the grid that represents boundary layer, elements in the shape of polyhedral prisms have been selected. They transit through buffer layers (including polyhedral elements) into cubical cells in the form of cuboids in the central part (core) of the pipeline.

The adopted boundary conditions determine movement inside the domain and the selection of correct parameters influences the process of calculations. For the conducted tests, a schema has been selected where the outlet plane is described with a condition *Pressure Outlet* (140 kPa of absolute pressure). The inlet plane to the measurement channel is described with a condition *Velocity Intel*, implementing planes with velocity profiles that correspond to the developed mass flows. Border walls of the models have been described by the parameter *wall* that does not consider wall roughness. The fluid adopted for numerical calculations is water with constant temperature of 20 °C, density at 998.2 kg/m^3^ and dynamic viscosity of 0.001003 kg/ms.

The values of flow rate of fluid flowing through the tested orifices has been selected in such a way that they coincide with the scope of obtained values of mass flow at the measuring station. Five velocity profiles have been created, corresponding to the following mass flow rates: 0.25 kg/s, 0.35 kg/s, 0.45 kg/s, 0.55 kg/s and 0.70 kg/s

The settings of the computational method were read in a standard configuration suitable for the Transition SST turbulnetic model using the Coupled scheme, with the double precision solver enabled. During the iteration, the value of the flux of the flowing fluid on the inlet and outlet planes was monitored. The condition for completing the iteration was for the residual monitor to reach a residual of 0.001 for the continuity and 5 model equations describing the Transition SST model.

### Numerical analysis of particle entrainment limit velocity

**Figure 2:**
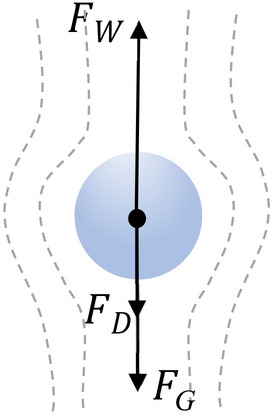
Forces acting on a spherical particle submerged in fluid [own work].

For the purpose analysis of the research problem it has been assumed that solid particles that form inclusions in the passing fluid are spherical in shape, with density ${\rho }_{cz}$ and diameter ${d}_{cz}$ (Fig. 2). For the purpose analysis of the research problem it has been assumed that solid particles that form inclusions in the passing fluid are spherical in shape, with density ${\rho }_{cz}$ and diameter ${d}_{cz}$ (Fig. 2). Using the Archimedes criterion number, a set of particles suspended in a liquid with density ${\rho }$ and dynamic viscosity was determined ${\mu }$. This set is described by the following equation:

17 $$Ar=\frac{g\left({\rho }-{\rho }_{cz}\right)\cdot {\rho }\cdot {{L}_{V}}^{3}}{{\mu }^{2}}$$

where ${L}_{V}$ is the ratio of spherical particle volume ${V}_{cz}$ to its total value ${P}_{{c}_{CZ}}$:

18 $${L}_{V}=\frac{{V}_{cz}}{{P}_{{c}_{CZ}}}=\frac{\frac{4}{3}\cdot \pi \cdot {{r}_{cz}}^{3}}{4\cdot \pi \cdot {{r}_{cz}}^{2}}=\frac{{d}_{cz}}{6}$$

After simplifications, the relationship of Archimedes' number (17) can be written in the form of Eq. (19) in which the parameters defining the size and mass of the inclusion particle, as well as the density and viscosity of the flowing fluid, are "sewn in”:

19 $$Ar=\frac{g\left({\rho }-{\rho }_{cz}\right)\cdot {\rho }\cdot {{d}_{cz}}^{3}}{216\cdot {\mu }^{2}}$$

Using this correlation of the physical properties of a particle (diameter ${d}_{cz}$ and density ${\rho }_{cz}$ suspended in a fluid characterized by dynamic viscosity $\mu$ and density $\rho$, the size of a particle suspended in a flowing stream of flowing fluid is defined in a dimensionless way.

A solid particle in the fluid can move on forced curvilinear paths resulting from velocity distribution as well as free falling/floating. Therefore, the manner of movement of particles in a certain area is dependent on the velocity of the fluid. To determine the phenomenon of self-cleaning of the measuring orifice from impurity particles (with a density ${\rho }_{cz}$ lower than the density of the fluid $\rho$) located in front of the measuring orifice, the test was limited only to velocities resulting from free falling/floating, depending on the density of the examined particle (${\rho }_{cz}$)^1,35^. Sense of vector of particle velocity is determined by the difference between fluid density ($\rho$) and density of a solid (${\rho }_{cz}$) submerged in the fluid. In a case when ${\rho }_{cz}-\rho <0$ the fluid displaces the submerged solid, therefore the velocity vector is sensed in the opposite direction to gravitational force.

The values of the velocity of a freely moving particle are calculated from the balance of the forces acting on the particle of a solid body immersed in a fluid according to Eq. (20) The values of the forces of gravity ${F}_{G}$, buoyancy force ${F}_{W}$ and drag force ${F}_{D}$ which are illustrated in the figure (Fig. 2) are described by equations:

20 $${F}_{G}={F}_{W}+{F}_{D}$$

where

21 $${F}_{G}={\rho }_{cz}\cdot g\cdot \frac{\pi \cdot {{d}_{cz}}^{3}}{6}$$

22 $${F}_{W}={\rho }\cdot g\cdot \frac{\pi \cdot {{d}_{cz}}^{3}}{6}$$

23 $${F}_{D}={C}_{D}\cdot \frac{\pi \cdot {{d}_{cz}}^{2}}{4}\cdot \rho \cdot \frac{{v}_{u}^{2}}{2}$$

By substituting the formulas for the individual components of ${F}_{G}$, ${F}_{W}$ and ${F}_{op}$, under the force balance Eq. (20), the equation was obtained:

24 $${C}_{D}*\frac{\pi *{{d}_{cz}}^{2}}{4}*\rho *\frac{{v}_{cz}^{2}}{2}=\left({\rho }_{cz}*g*\frac{\pi *{{d}_{cz}}^{3}}{6}\right)-\left({\rho }*g*\frac{\pi *{{d}_{cz}}^{3}}{6}\right)$$

The drag coefficient ${C}_{D}$ existing in Eq. (24) is a function of number ${Re}_{cz}.$ Therefore, it is dependent on the diameter of particle ${d}_{cz}$ and its floating velocity ${v}_{u}$ as a result of free, unbounded movement^36^. The Reynolds number was calculated from the relationship (25):

25 $${Re}_{cz}=\frac{{\vartheta }_{u}*{d}_{cz}}{\nu }$$

Table 1 shows the equations for determining the *C*_*D*_ value depending on the Reynolds number ${Re}_{cz}$ value for the flowing particle.

**Table 1:** Fields of application and value of drag coefficients ${C}_{D}$ from ${Re}_{cz}$^37^.

	${Re}_{cz}$	${C}_{D}$
Laminar movement according to Stokes' law	${Re}_{cz}\le 0.3$	$\frac{24}{{Re}_{cz}}$
Transitional movement in accordance with Allen's law	${0.3<Re}_{cz}\le 5$	$\frac{\mathrm{26,5}}{{Re}_{cz}^{\mathrm{0,88}}}$
	${5<Re}_{cz}\le 100$	$\frac{\mathrm{18,6}}{{Re}_{cz}^{\mathrm{0,64}}}$
Turbulent motion according to Newton's law	${100<Re}_{cz}\le 2*{10}^{4}$	$\frac{\mathrm{18,6}}{{Re}_{cz}^{\mathrm{0,64}}}+\frac{4}{9}*\frac{{Re}_{cz}^{\mathrm{0,8}}}{330+{Re}_{cz}^{\mathrm{0,8}}}$
	${Re}_{cz}>2*{10}^{4}$	$\frac{4}{9}$

After rearranging the force balance Eq. (24), the value of particle velocity moving freely in the fluid has been determined in the following form:

26 $${v}_{u}=\sqrt{\frac{4\cdot ({\rho -{\rho }_{cz}})\cdot g\cdot {{{d}_{cz}}}}{3\cdot {C}_{D}\cdot \rho }}$$

When calculating the lift velocity ${v}_{u}$, one should perform a sequence of looped calculations, and for the first approximation, the value of the resistant coefficient ${C}_{D}=1$ should be taken. From the obtained value of the lift velocity, the value of the resistant coefficient ${C}_{D}$ based on Table 1 should be determined.

By generating a graph of the function of the criterion numbers $Ar=f({Re}_{cz})$, one obtains a set of points forming the limiting curve of the particles that are carried away and float in the fluid. Knowing the equations determining the Archimedes number (19) and Reynolds number (25) of a particle, it is possible to determine the minimum fluid velocity that allows the floating particles to be carried away and transported through the segmental orifice and segmental orifice with an inclined inflow plane.

From the CFD numerical simulations performed, vector maps of the velocity of the flowing fluid through the measuring orifice under study were generated. They define the area upstream of the measuring orifice bounded by the pipeline plane, the inflow plane of the measuring orifice and a boundary line composed of points where the fluid velocity $v$ is less than the calculated velocity that allows to carry away the particles ${v}_{u}$. By inclining the inflow plane of the segmented orifice, a new velocity distribution is created, with a decreasing area in which the fluid has a velocity $0\le v{\le v}_{u}$. With an increase in the velocity of the flowing fluid (Reynolds number increases), the area of accumulation of particles in front of the measuring orifice is reduced by their entrainment by the flowing fluid stream.

## Results and discussion

To create a computational grid in a 3D model a volume closed inside the measurement pipeline has been adopted. The pipeline is composed of the tested measurement orifice and straight segments before and after the measurement orifice. It has been assumed the length of the inlet segment before the orifice will be 175 mm (3.5·D) long, and the outlet segment behind the orifice will be 425 mm (8.5·D) long.

### Selecting numerical grid and turbulent model

**Figure 3:**
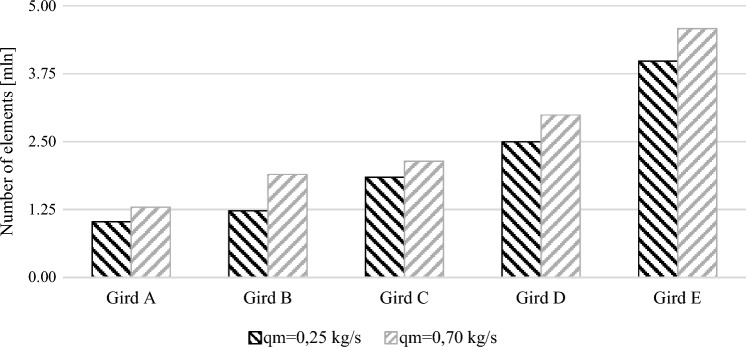
The size of the tested grids [own work].

The selection of numerical grid and turbulent model was made using a 3D model of the measurement pipeline with a segmental orifice with module $m=0.102$. The tests were conducted for velocity profiles corresponding to mass flow 0.25 kg/s and 0.70 kg/s. 10 grids were generated in total (5 grid sizes for each defined mass flow) with refinement in the boundary layer. The grids have been marked with letters in alphabetical order—from the smallest A (ca. 1 million elements) to the largest E (ca. 4.5 million elements). The sizes are presented in Fig. 3.

**Figure 4:**
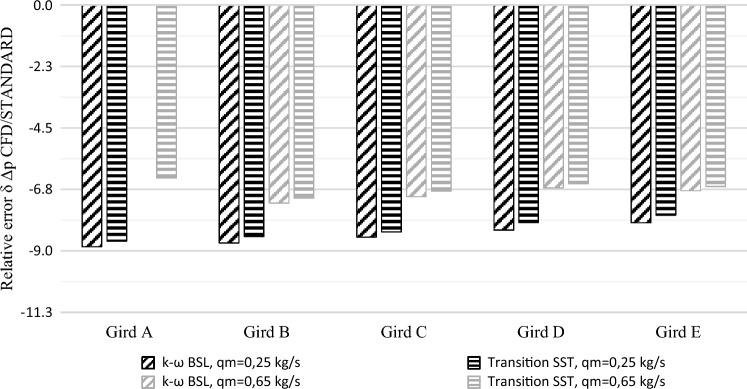
Results comparison of the tested numerical grids and turbulent models [own work].

Simulation calculations have been made for all test grids (Fig. 4) using two turbulent models $k-\omega BSL$ and $TransitionSST$. On this basis, swelling values on the segmental orifice $\Delta {p}_{CFD}$ have been determined for selected mass flows. In order to compare the value for differential pressure obtained from the CFD simulation with the theoretical value, engineering calculations of pressure build-up ${\Delta {p}}_{PN}$ have been made according to standard^17^ using the Eq. (27):

27 $${\Delta p}_{PN}=\frac{8\cdot {{q}_{m}}^{2}\cdot \left(1-{m}^{2}\right)}{{\pi }^{2}\cdot {C}^{2}\cdot {m}^{2}\cdot {D}^{4}\cdot \rho }$$

In this equation, the value of mass flow ${q}_{m}$ is represented by a value in boundary conditions of numerical simulations as ${{q}_{m}}_{CFD}$ in the form of defined velocity profiles. Relative error of the obtained differential pressure on measurement orifice ${\delta }_{{\Delta {p}_{CFD}}}$ has been calculated. Relative error ${\delta }_{{\Delta {p}_{CFD}}}$ of comparison of swelling pressure has been calculated from the following dependence (28):

28 $${\delta }_{\Delta {p}_{CFD}}=\frac{{\Delta {p}_{CFD}}-{{\Delta {p}_{PN}}}}{{{\Delta {p}_{PN}}}}\cdot 100[\%]$$

where: $\Delta {p}_{PN}$ is the value of differential pressure on the measurement orifice, calculated according to standard^17^. $\Delta {p}_{CFD}$ is the value of differential pressure determined from CFD numerical simulation.

The results obtained from the conducted validation were presented in the form of a graph (Fig. 4). When it comes to grid A with a specified mass flow ${q}_{m}=0.70$ kg/s and turbulent model *k*–*ωBSL* numerical calculations were not completed in the iteration process. The obtained relative error for the same computational grid with the same specified mass flow for model $TransitionSST$ deviates from the results and trend of the other tested grids. For this reason grid A was rejected from further considerations.

**Figure 5:**
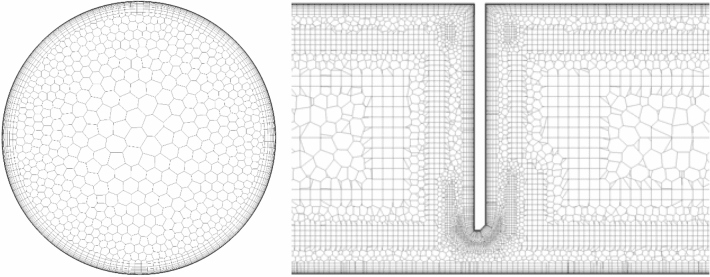
The structure of the numerical grid for segmental orifice with module $m=0.102$ for mass flow ${q}_{m}=0.70kg/s$ [own work].

With acceptable error of simulation and time of numerical calculations, computational grid C has been selected. It is composed of 2.14 million elements for flow ${q}_{m}=0.70$ kg/s and 1.84 million elements for flow ${q}_{m}=0.25$ kg/s Figure 5 presents a part of the selected grid in a cross-section and longitudinal section of the pipeline in the measurement orifice area.

### Flow charts of the tested measurement orifice

Data collected from the conducted experimental tests and CFD simulations was used to create charts and graphs that represent the tested flow parameters of the segmental orifice and the segmental orifice with inclined inflow plane. Table 2 presents the values of differential pressure ${\Delta p}_{CFD}$ from CFD numerical simulation, obtained on a measurement orifice with specified mass flows ${q}_{{m}_{CFD}}$. Simulations were conducted according to the established boundary conditions, using computational grid C and turbulent model $TransitionSST$.

**Table 2:** Vaules $\Delta p$ from CFD numerical simulations for segmental orifice and segmental orifice with inflow plane inclined by angle α [own work].

${q}_{{m}_{CFD}}$ (kg/s)	Re (–)	Segmental orifice	$\alpha =80^\circ$	$\alpha =70^\circ$	$\alpha =60^\circ$
		${\Delta p}_{CFD}$ (Pa)	${\Delta p}_{CFD}$ (Pa)	${\Delta p}_{CFD}$ (Pa)	${\Delta p}_{CFD}$ (Pa)
0.2494	6288	1865	1732	1593	1449
0.3491	8801	3705	3432	3146	2863
0.4489	11,318	6090	5667	5194	4706
0.5486	13,832	9140	8482	7779	7053
0.6984	17,607	14,867	13,780	12,654	11,462

Experimental tests were conducted according to assumptions presented earlier. Apart from measurement of swelling (pressure difference on the measurement element) for individual flow rates of the fluid passing through the measurement pipeline, fluid temperature was also recorded before and after the series of measurements. For further calculations, arithmetic means from measurement series were used. Tables 3 and 4 compile data obtained from experimental tests and extended type B relative uncertainties of flow ${q}_{m}$ and differential pressure $\Delta {p}$ measurements.

**Table 3:** Values obtained from experimental measurements for segmental orifice [own work].

${q}_{V}$ (dm^3^/s)	$\Delta p$ (Pa)	$T$ (°C)	$\rho$ (kg/m^3^)	${q}_{m}$ (kg/s)	$Re$ (–)	$U({q}_{m})/{q}_{m}$ (%)	$U(\Delta p)/\Delta p$ (%)
0.2491	1977	20.80	998.28	0.2487	6316	0.4629	1.1228
0.3313	3489	21.05	998.21	0.3307	8451	0.4053	0.6363
0.3913	4862	21.35	998.13	0.3906	10,054	0.3785	0.4565
0.4461	6316	21.70	998.03	0.4452	11,556	0.3603	0.3514
0.4774	7237	22.00	997.95	0.4765	12,456	0.3518	0.3067
0.5135	8372	22.20	997.90	0.5124	13,460	0.3433	0.2651
0.5438	9387	22.40	997.84	0.5426	14,320	0.3370	0.2365
0.5698	10,309	22.55	997.80	0.5685	15,058	0.3322	0.2153
0.5992	11,401	22.65	997.77	0.5978	15,871	0.3272	0.1947
0.6227	12,313	22.60	997.79	0.6213	16,474	0.3235	0.1803

**Table 4:** Values obtained from experimental measurements for segmental orifice with inclined plane ($\alpha =70^\circ )$ [own work].

${q}_{V}$ (dm^3^/s)	$\Delta p$ (Pa)	$T$ (°C)	$\rho$ (kg/m^3^)	${q}_{m}$ (kg/s)	$Re$ (–)	$U({q}_{m})/{q}_{m}$ (%)	$U(\Delta p)/\Delta p$ (%)
0.2493	1599	20.95	998.24	0.2489	6345	0.4627	1.3883
0.3327	2856	21.15	998.18	0.3321	8509	0.4045	0.7771
0.3938	4000	21.15	998.18	0.3930	10,068	0.3776	0.5549
0.4526	5283	21.35	998.13	0.4518	11,629	0.3585	0.4202
0.4863	6090	21.55	998.07	0.4853	12,553	0.3496	0.3645
0.5204	6966	21.65	998.05	0.5194	13,467	0.3418	0.3187
0.5512	7799	21.75	998.02	0.5501	14,296	0.3356	0.2846
0.5778	8557	21.85	997.99	0.5766	15,021	0.3308	0.2594
0.6083	9477	21.90	997.98	0.6071	15,833	0.3258	0.2342
0.6328	10,253	21.75	998.02	0.6315	16,413	0.3221	0.2165

### Validation of computational CFD simulation based on experimental data

**Figure 6:**
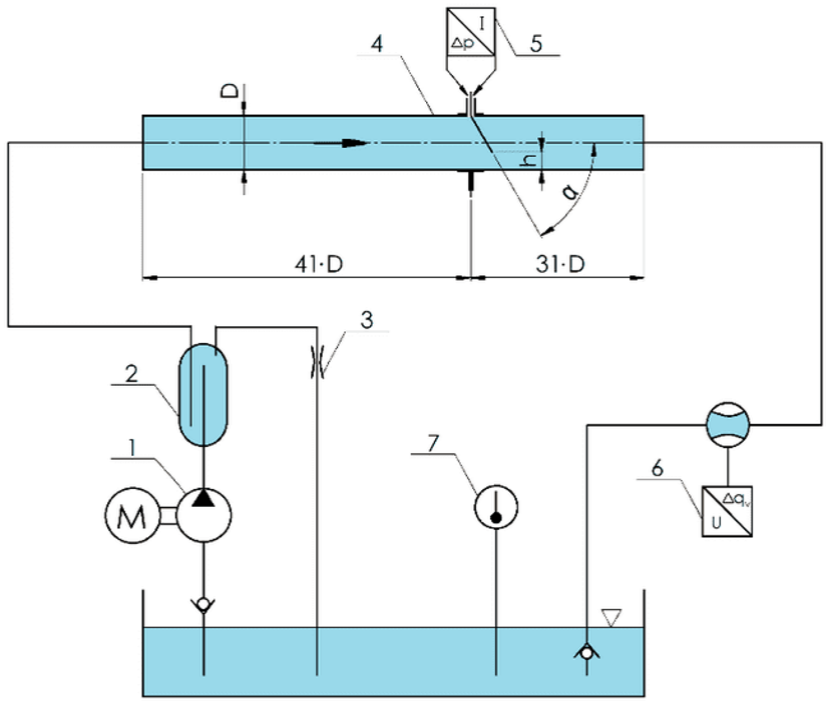
Measurement station—block diagram [own work].

In a hydraulic measurement station (Fig. 6), using an eccentric pump with constant rate of delivery (1), the fluid in the measurement unit is moved from the main tank with absolute pressure at ca. $140$ kPa. Before pumping the fluid into the measurement system, the fluid enters a deaerating vessel (2). Forced by the pump, the fluid flows out of the bottom port (2a) whose end is located inside the tank, at $3/4$ of its height from the bottom tank end. In the top tank end there is a port (2b) from which fluid flow with possible air bubbles exits, via adjustable side bleed, through replaceable glands (3). This allows for gradual adjustment of the measurement volume flow within the range of $0.25{{\text{dm}}}^{3}/{\text{s}}<{q}_{V}<0.65{{\text{dm}}}^{3}/{\text{s}}$. The final outlet port (2c) is also located in the top tank end but it is submerged inside the deaerator at the depth of ca. $3/4$ of the entire vessel height. This allows for collecting fluid with no air bubbles and forcing it into the hydraulic measurement station. The fluid, separated from possible gas fractions, flows through the measurement pipeline (4) and then returns to the main tank, creating a closed system of fluid flow.

**Figure 7:**
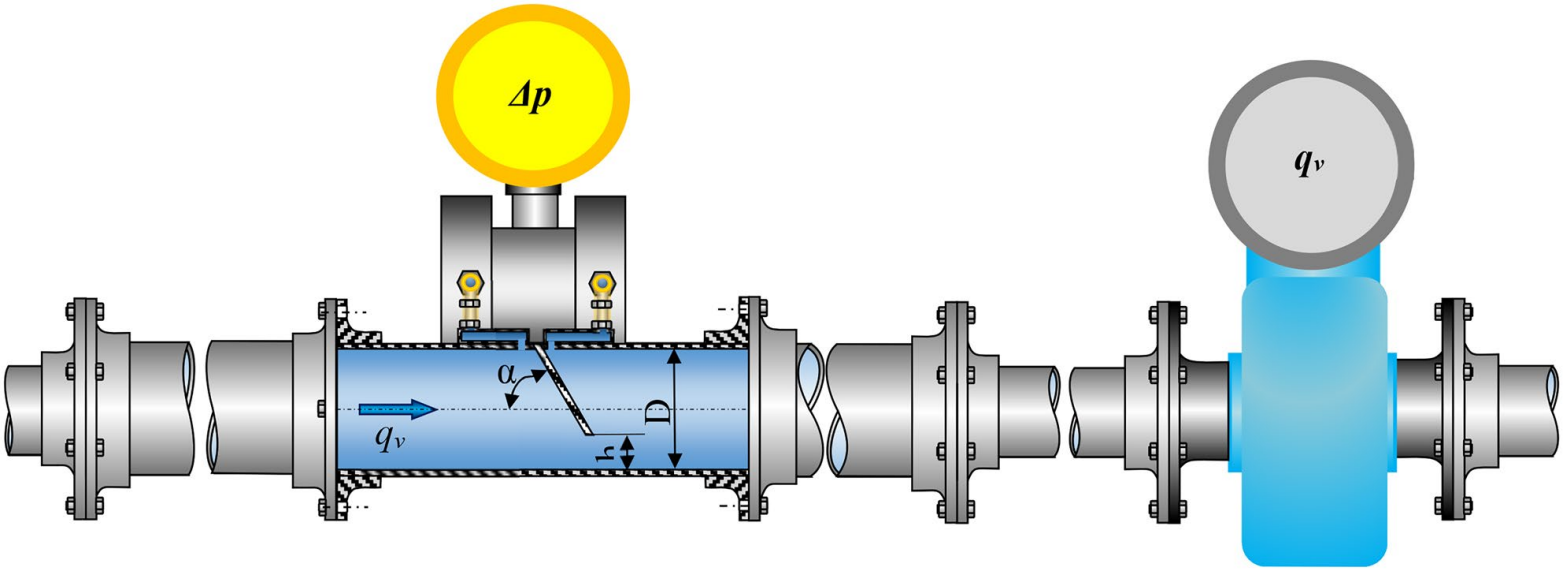
Measurement system of a segmental flow meter with a flow meter acting as standard [own work].

The measurement pipeline (Fig. 7) is composed of reducing tested flow meter, connected to a differential pressure transducer (5), electromagnetic flow meter acting as standard (6), and straight segments made of stainless steel, with internal diameter of *D* = 50 mm. The segment before the tested flow meter with segmental orifice is 2.05 m ($41\cdot D$) in length, and the one behind the measurement orifice is 1.55 m ($31\cdot D$)—both lengths remain within the scope recommended by source literature^20^. In order to retain axial symmetry when replacing the tested flow meter with a skew segmental orifice and straight segments of the pipeline at the point of connection, fitting flanges with a centering lock have been used.

In order to determine the dependence of function ${q}_{v}=f(\Delta p)$ simultaneous measurements must be made for the value of swelling $\Delta p$ obtained on the tested orifice and the flow ${q}_{v}$ of fluid flowing through it at the same time.

The static pressure of fluid on the tested segmental orifice was measured at pressure input points located at the corner, on the opposite side of the flow-through opening, in the top part of the flow meter. Differences between high pressure area (before the orifice) and low pressure area (behind the orifice) were measured with a programmable APR-2000/ALW differential pressure transducer with an output current signal from 4 to 20 mA. The transducer for tests was programmed for measurement range of $\Delta p=12.8$ kPa with time constant of $t=5$ s with a limiting error $\Delta p=0.15\%.$ The value of volume flow of the fluid passing through the tested orifice was measured with a PROMAG 30AT15 electromagnetic flow meter with output current signal from 4 to 20 mA. It is located behind the tested flow meter in a parallel DN15 pipeline. Its measuring range ${q}_{v}$ was set to $3.6$ m^3^/h with the same time constant $t=5$ s. The flow meter has a limiting error $\Delta {q}_{v}=\pm (0.2\%\cdot {q}_{{v}_{mier}})\pm 0.05\%\cdot {q}_{v}$, and was used as standard in the measurement system. Fluid temperature was measured with an electronic thermometer graduated in intervals of $0.1$ °C after prior calibration with standard laboratory thermometer. Temperature measurement was registered (recorded) at the beginning and end of each individual measurement series—arithmetic mean was considered in calculations. Temperature value is essential for determining fluid density, while determining mass flow and kinematic viscosity for calculating Reynolds number.

**Figure 8:**
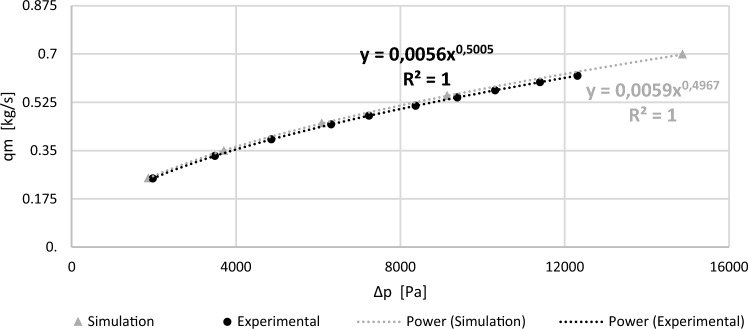
Flow chart for a segmental orifice with module $m=0.102$ [own work].

**Figure 9:**
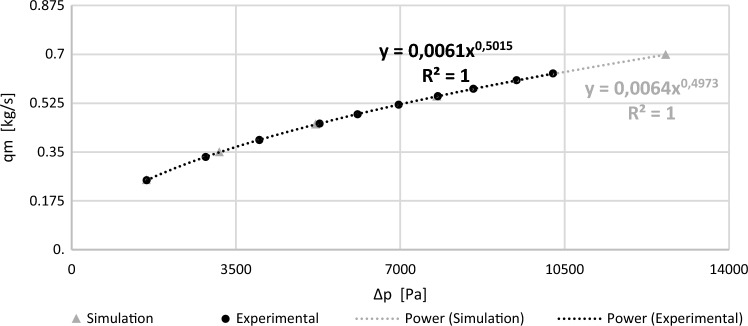
Flow chart of a segmental orifice with inflow inclined by angle $\alpha =70^\circ$ and module $m=0.102$ [own work].

Flow charts have been presented in the form of graphs. Figure 8 presents flow charts obtained from CFD experimental tests and simulations for a segmental orifice, while Fig. 9 presents the same for a segmental orifice with plane inclined by angle $\alpha =70^\circ$. Power trend lines have been plotted on the graphs for ${q}_{m}=f(\Delta p)$, obtaining their equations and alignment coefficients ${R}^{2}$.

### Maps of velocity and static pressure distribution

**Figure 10:**
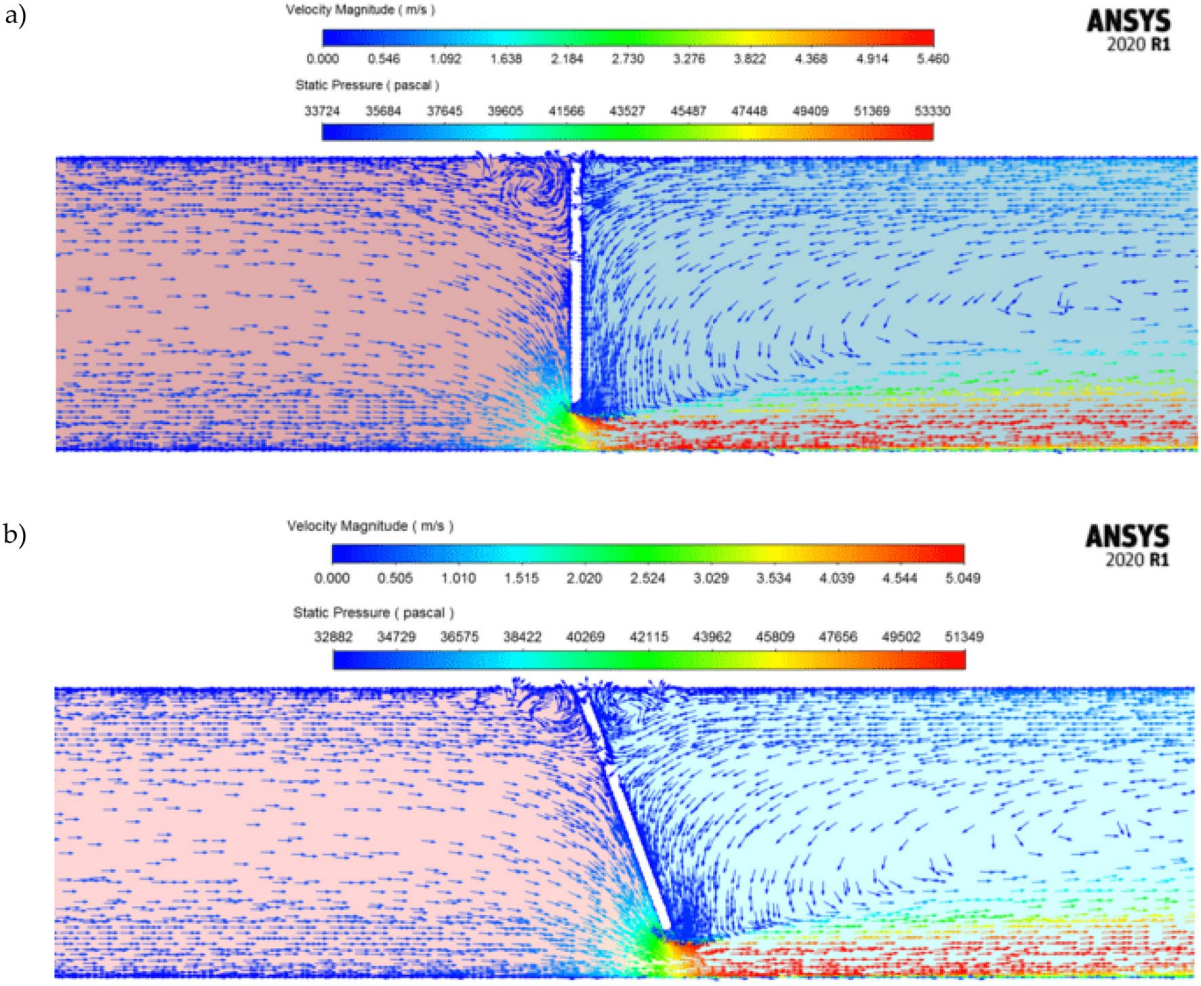
Static pressure distribution with velocity vectors for mass flow ${q}_{m}=0.7$ kg/s: (**a**) segmental orifice, (**b**) segmental orifice with inclined plane [own work].

Figure 10 presents a graphic image of results from numerical simulations for a segmental orifice ($\alpha =90^\circ )$ and a segmental orifice with inclined downflow plane $(\alpha =70^\circ )$ with passing mass flow ${q}_{m}=0.70$ kg/s. Static pressure distribution before and after the orifice is presented as a spectrum of colors and velocity distribution as a vector spectrum of colors.

### Determining the blind spot reduction coefficient

**Figure 11:**
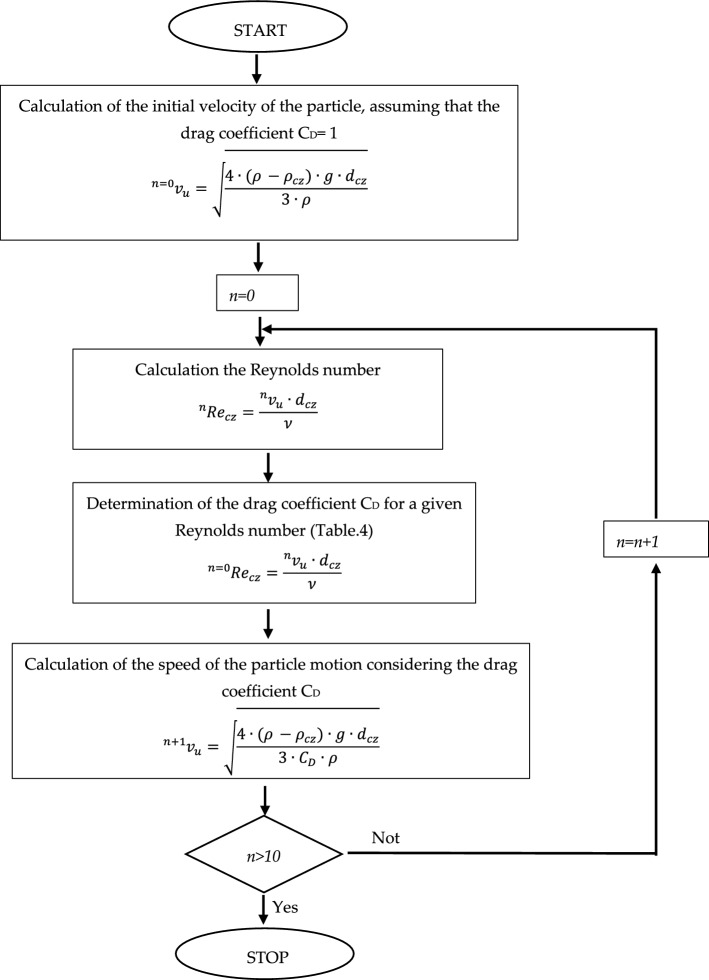
Diagram of the process of approximating particle movement velocity [own work].

Having analyzed the influence of inflow plane inclination, an example set of solids has been determined which contains solids whose particle diameter remains within a range of ${d}_{cz}=0.2\dots 1$ mm with density ${\rho }_{cz}$ from a range $700\dots 950$ kg/m^3^ submerged in a fluid with parameters consistent with the fluid used in numerical calculations ($\rho =998.2$ kg/m^3^,  $\mu =10.3\times {10}^{-4}$ Pa s). Using Eq. (20) for this data, velocity value ${v}_{u}$ for a particle was calculated, and then the Reynolds number ${Re}_{cz}$. When determining the value of floating speed ${v}_{u}$ a series of 10 computational iterations was conducted, approximating the value of floating velocity ${v}_{u}$. When calculating the first approximation of floating velocity ${v}_{u}$ the influence of drag coefficient was omitted and value ${C}_{D}=1$ was assumed, resulting in obtaining input value of floating velocity. On the basis of the paper^36^, where the most popular methods of determining the resistance coefficient are discussed, the relationships presented in Table 1^37^. Based on the Reynolds number of the particle ${Re}_{cz}$ the value of drag coefficient ${C}_{D}$ was determined from a function. When determining the value of floating speed ${v}_{u}$ a series of 10 computational iterations was conducted, approximating the value of floating velocity ${v}_{u}$. The calculation algorithm has been presented in the form of a diagram (Fig. 11).

**Figure 12:**
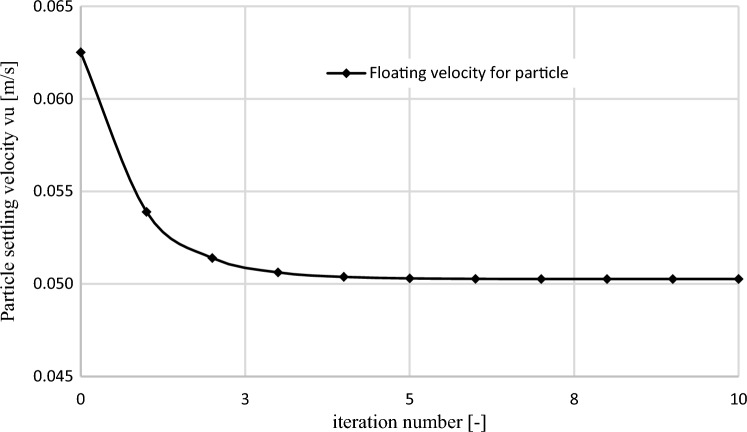
The course of the obtained values of floating velocity for a particle with Archimedes number Ar = 12.74 in the process of iteration [own work].

The graph in Fig. 12 presents the value of floating velocity ${v}_{u}$ obtained in each following iteration during the conducted calculations for a particle with $Ar=12.74$ number (Eq. 19). The horizontal axis is marked with subsequent numbers of calculation loop (iteration) where zero denotes the value of particle velocity with drag coefficient *C*_*D*_ = 1.

**Figure 13:**
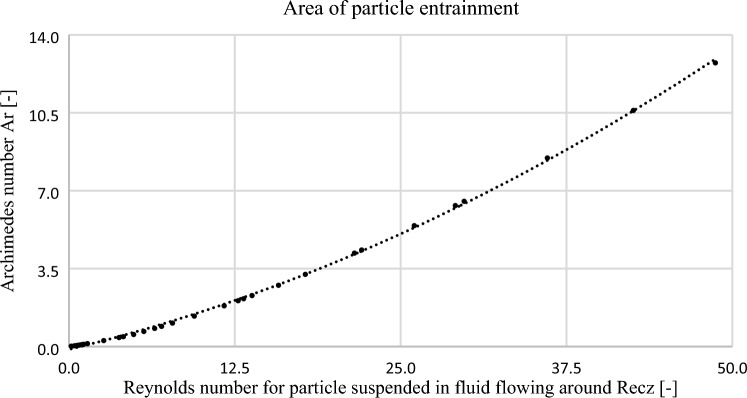
Boundary value of the Reynolds number where particles described with Archimedes number are entrained [own work].

The graph in Fig. 13 presents a set of point described with characteristic numbers function $Ar=f({Re}_{cz})$. The points form a limit curve of entrainment of particles floating in the fluid. Knowing the equations that determine the Archimedes number (19) and Reynolds number (25) of the particle, it is possible to determine the minimum velocity of fluid which allows for entrainment and transport of particles floating in the fluid through the segmental orifice and segmental orifice with inclined inflow plane.

**Figure 14:**
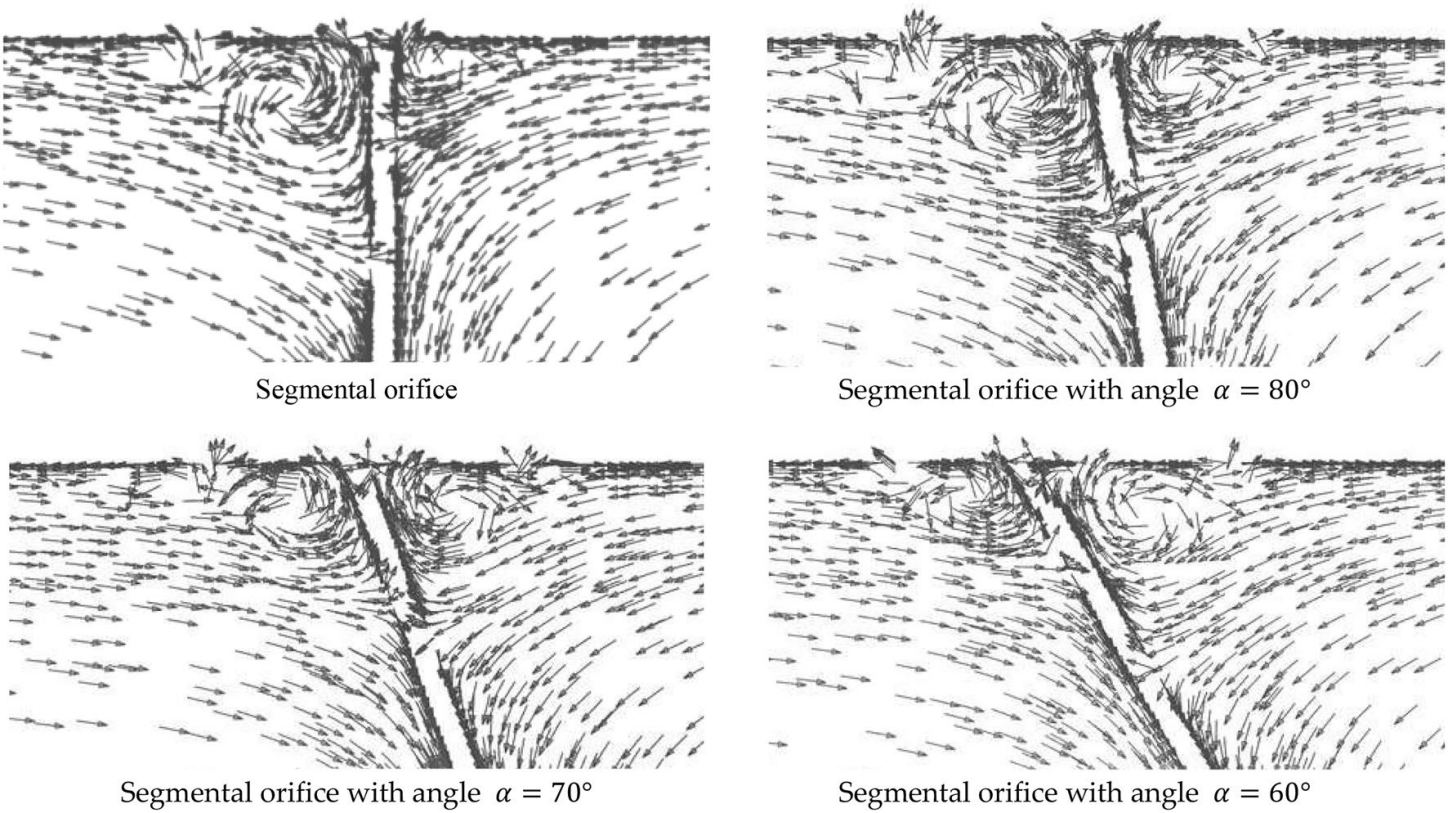
Area of vortex occurrence before the plane of the segmental orifice for flow ${q}_{m}=0.70$ kg/s [own work].

Considering the calculated values of solid particle floating velocity (26), CFD numerical simulations have been used to determine areas of deposits ‘stockpiling’. The velocity of solid particle entrainment that occurs in this area is lower than floating velocity. The areas were being determined on the basis of vectorial distributions of velocity in the passing flow presented as an example in Fig. 14 for a passing flow ${q}_{m}=0.70$ kg/s with Archimedes number $Ar=12.74$. Before the orifice plane, in its upper part, additional local vortexes occur (increase in fluid velocity) that entrain particles, but keep them in the area where the vortexes occur. Therefore, the surface of the vortexes was also added to the area of deposits ‘stockpiling’. The illustrations show that when the downflow plane of the segmental orifice is inclined, both the size of the occurring vortex and the area of the deposit decrease.

The conducted CFD numerical simulations of velocity distributions were used to determine areas ${A}_{\alpha^\circ }$ where velocity values of the passing fluid $v$ remain within range $0\le v{\le v}_{u}$ for a segmental orifice (${A}_{\alpha =90^\circ })$ and segmental orifice with inclined inflow plane α: 80°, 70° and 60° (${A}_{\alpha =n^\circ })$. Based on the obtained areas ${A}_{\alpha =90^\circ }$ and ${A}_{\alpha =n^\circ }$ relative percentage reduction of ‘stockpiling’ area was determined for a segmental orifice with a plane, in relation to a segmental plane. The reduction has been described with the Eq. (29).

29 $${\psi }_{\alpha =n^\circ }=\frac{{A}_{\alpha =90^\circ }{-A}_{\alpha =n^\circ }}{{A}_{\alpha =90^\circ }}\cdot 100[\%]$$

This relation was acknowledged as quality index (${\psi }_{\alpha =n^\circ }$) of ‘self-purging’ of the segmental orifice as a result of inclining the inflow plane. The obtained area values are presented in Table 5, depending on the flow size of the passing fluid defined by Reynolds number.

**Table 5:** Index ${\psi }_{\alpha =n^\circ }$ for measurement orifices with module $m=0.102$ [own work].

*Re* (–)	${A}_{\alpha =0^\circ }$ (mm^2^)	${A}_{\alpha =80^\circ }$ (mm^2^)	${\psi }_{\alpha =80^\circ }$ (%)	${A}_{\alpha =70^\circ }$ (mm^2^)	${\psi }_{\alpha =70^\circ }$ (%)	${A}_{\alpha =60^\circ }$ (mm^2^)	${\psi }_{\alpha =60^\circ }$ (%)
6290	410	351	14.30	277	32.40	231	43.63
8800	346	274	20.85	222	35.68	168	51.54
11,320	312	251	19.61	192	38.39	145	53.63
13,830	290	224	22.62	173	40.29	128	55.92
17,610	260	203	21.74	150	42.32	113	56.37

**Figure 15:**
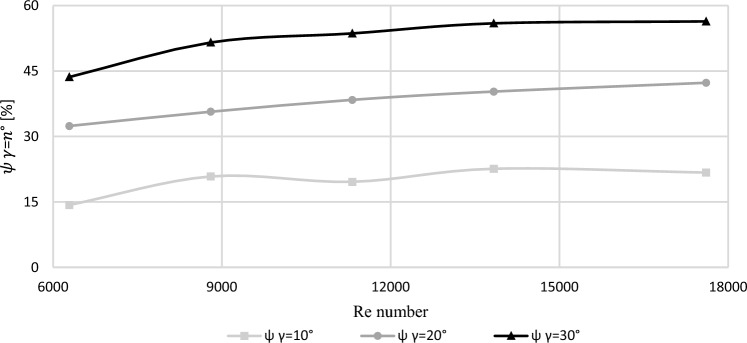
The value of purging index ${\psi }_{\alpha =n^\circ }$ of the tested segmental orifices with module $m=0.102$ [own work].

The values of purging index ${\psi }_{\alpha =n^\circ }$ obtained from the calculations in a function of the number Re for module $m=0.102$ have been presented in the form of a graph (Fig. 15).

## Conclusion

The article has presented the results of simulation tests in Ansys Fluent 2020R1 software as well as experimental tests on two orifices (a segmental orifice and a segmental orifice with downflow plane inclined by angle *α*) with module $m=0.102$ in a DN50 pipeline. Scientific research has been conducted within the range of Reynolds number 6300 < Re < 17600 (area of developing turbulent flow).

Before conducting target CFD numerical calculations, an analysis was conducted of matching two turbulence models: *k*–*ωBSL* and $TransitionSST$, for theoretical calculations for a segmental orifice with module *m* = 0.102 ($\alpha =90$°)^17^. Out of the two models, the $TransitionSST$ model was characterized by smaller matching errors, with extreme mass flows ($0.25$ kg/s and $0.70$ kg/s) adopted for calculations.

For this reason, the turbulent model $TransitionSST$ with a grid of 2.14 million elements was selected for further CFD numerical calculations. With acceptable error and time of numerical calculations, it allowed for accurate reflection of physical phenomena occurring in a passing fluid flow.

The obtained simulation results are convergent with conducted experimental tests for the segmental orifice ($\alpha =90^\circ$) as well as orifice with inclined downflow plane ($\alpha =70^\circ$) with module $m=0.102$. Matching has also been achieved between points and power trend lines characterized by coefficient *R*^*2*^ equal to one.

In the proposed solution, the occurring inclinations of the orifice plane causes a stepless, smooth transition from circular cross-section of the pipeline to the area of flow-through opening in the shape of circular sector. When inclination angle $\alpha$ is reduced, the length orifice plane increases, reducing the flow section. Therefore, the measurement value of swelling pressure on the orifice decreases, and, consequently, so does constant pressure loss.

The analysis involved the possibility of segmental orifice self-purging from solid particles suspended in the passing fluid $({\rho }_{cz}-\rho <0)$, based on numerical simulations. For this purpose, calculations for spherical solid particles were made, determining the boundary line between particle floating and entrainment by the flow, based on non-dimensional numbers: the Archimedes number and the Reynolds number (Fig. 13).

The article presented a method to reduce the area of ‘stockpiled’ deposits (concentration of floating solid particles in fluid) before the segmental orifice, by inclining its downfall plane. As the inclination angle of the segmental orifice inflow plane increases, the area of ‘stockpiled’ deposits decreases. For an inclination angle $\alpha =60^\circ$ with number Re ˃ 8800 it decreases by more than 50% compared to a segmental orifice with angle $\alpha =90^\circ$.

As can be seen, the inclination of the downflow plane causes entrainment of a much larger amount of floating particles, reducing the area of ‘stockpiled’ deposits, which improves kinematics of the passing fluid flow.

Further research is planned which will allow to optimize the inclination angle of the segmental orifice downflow plane due to the occurrence of a similar deposit ‘stockpiling’ area on the outflow side of the orifice.

## Data Availability

The datasets used during the current study are available from the corresponding author on reasonable request.

## List of symbols

*Ar*: Archimedes number (–)
*A*_*γ°*_: Surface area (mm^2^)
$${C}_{D}$$: Drag coefficient (°)
$D$: Pipeline internal diameter (m)
${d}_{cz}$: Particle diameter (sphere) (m)
$${F}_{D}$$: Drag force (N)
$${F}_{G}$$: Gravity force (N)
$${F}_{W}$$: Displacement force (N)
${F}_{1}$: Cross-sectional area of the pipeline (m^2^)
${F}_{2}$: Area of the measurement orifice opening (m^2^)
*g*: Gravitational acceleration (m/s^2^)
*h*: Orifice constrictions (m)
$${L}_{V}$$: Characteristic dimension (–)
*m*: Orifice module (–)
${V}_{cz}$: Particle (sphere) volume (m^3^)
${P}_{{c}_{CZ}}$: Particle (sphere) surface area (m^3^)
*p*: Static pressure (Pa)
*q*_*m*_: Mass flow (kg/s)
${q}_{v}$: Volume flux (dm^3^/s)
*Re*: Liczba Reynoldsa (–)

## Greek letters

$\gamma^\circ$: Orifice plane inclination angle (°)
$\mu$: Dynamic viscosity coefficient (Pa s)
${v}_{u}$: Particle floating velocity (m/s)
${v}_{1}$: Average velocity of the flowing fluid in the pipeline cross-section (m/s)
${v}_{2}$: Average velocity of the flowing fluid in the cross-section of the orifice opening (m/s)
$\rho$: Fluid density (kg/m^3^)
${\rho }_{cz}$: Solid density (kg/m^3^)
$${\mu }_{t}$$: Turbulent viscosity (m^2^/s)
*Δp*: Differential pressure (Pa)
${\psi }_{\gamma^\circ =n^\circ }$: ‘Self-purging’ quality index (%)

## Symbols in CFD simulations

${\Gamma }_{k}$, ${\Gamma }_{\omega }$: Effective diffusivity coefficients
${G}_{k}$: Coefficient of generating turbulence kinetic energy as a result of mean velocity gradients
${G}_{\omega }$: Coefficient of generating dissipation velocity in the transport of turbulence kinematic energy
${Y}_{k}$: Coefficient of dissipation of turbulence kinetic energy
${Y}_{\omega }$: Coefficient of dissipation in the transport of turbulence kinematic energy
${G}_{b}$,${G}_{\omega b}$: Coefficient of displacement effect in turbulence kinematic energy transport equation
${S}_{k}$, ${S}_{\omega }$: Constant definable coefficients
${G}_{k}^{*}$, ${Y}_{k}^{*}$: Derivative coefficients ${G}_{k}$ and ${Y}_{k}$ caused by separation
${P}_{\gamma 1},{E}_{\gamma 1}$, ${P}_{\gamma 2}$, ${E}_{\gamma 2}$: Coefficients of transition source, discontinuity destruction/relaminarization
${\widetilde{Re}}_{\theta t}$: Constant coefficient in model (Reynolds number)
